# Supplementary material for: Portuguese Lipid Study (e_LIPID)
Source: J Clin Med. 2024 Nov 19;13(22):6965. doi: 10.3390/jcm13226965 (PMC11595309; doi:10.3390/jcm13226965)
Supplement: Supplementary file 1 [file jcm-13-06965-s001.zip › jcm-2993831-supplementary.pdf]

Supplementary Data

Supplementary Table S1. 95% confidence intervals for percentile estimation in the Portuguese population for A) TC, B) LDL-C, C) HDL-C, D) TG, E) Lp(a), F) apoB, G) apoA1, H) non-HDL-C, I) sdLDL-C and J) VLDL.

**A) TC (mg/dl)**							
**Men**	5th	10th	25th	50th	75th	90th	95th
18-29 years	118.0 - 132.9	125.7 - 136.9	137.0 - 156.8	161.0 - 174.5	177.2 - 200.0	200.2 - 219.2	207.4 - 237.0
30-39 years	129.7 - 154.5	141.0 - 165.5	165.2 - 179.8	185.5 - 203.0	210.0 - 232.0	232.0 - 250.0	239.1 - 271.0
40-49 years	138.0 - 167.8	160.0 - 177.0	177.0 - 194.0	198.0 - 216.0	221.5 - 236.0	236.1 - 267.0	252.0 - 283.0
50-59 years	146.0 - 172.8	159.0 - 176.9	177.0 - 184.0	187.5 - 209.0	222.0 - 250.5	250.7 - 277.1	260.8 - 293.7
60-69 years	141.0 - 170.8	162.7 - 172.0	172.0 - 186.8	191.0 - 207.0	215.5 - 238.2	239.0 - 275.0	251.1 - 282.0
18-69 years	132.9 - 146.0	146.0 - 158.9	169.0 - 176.0	189.0 - 197.0	217.0 - 226.8	240.0 - 252.0	254.1 - 274.1
**Women**							
18-29 years	116.0 - 134.0	124.0 - 144.8	134.0 - 152.7	152.2 - 165.0	171.0 - 187.0	190.0 - 211.0	211.1 - 246.0
30-39 years	104.0 - 144.8	139.3 - 151.0	144.9 - 156.0	156.0 - 174.8	180.0 - 190.0	194.0 - 214.0	214.0 - 239.5
40-49 years	138.0 - 151.0	149.0 - 157.5	151.0 - 164.0	164.0 - 179.0	184.0 - 199.5	208.0 - 222.0	222.2 - 267.0
50-59 years	94.0 - 160.3	146.0 - 164.8	160.4 - 170.0	171.5 - 192.2	200.0 - 210.5	214.2 - 226.2	226.2 - 253.1
60-69 years	147.0 - 176.7	174.3 - 181.0	176.9 - 192.0	192.0 - 207.8	209.0 - 230.0	235.0 - 247.0	247.3 - 277.0
18-69 years	104.0 - 138.0	144.0 - 151.0	151.0 - 157.0	169.0 - 176.0	191.0 - 199.5	215.0 - 221.0	239.0 - 250.0
**Men + Women**							
18-69 years	138.0 - 147.0	151.0 - 156.0	170.0 - 175.0	191.0 - 197.0	217.0 - 222.2	241.0 - 250.2	255.0 - 267.1

**B) LDL-C (mg/dl)**							
**Men**	5th	10th	25th	50th	75th	90th	95th
18-29 years	56.0 - 69.7	60.8 - 76.8	76.8 - 92.0	97.0 - 110.0	115.0 - 131.0	131.1 - 143.3	136.2 - 151.6
30-39 years	75.9 - 92.7	81.0 - 99.7	99.8 - 117.5	122.5 - 133.0	137.5 - 155.0	155.0 - 177.1	163.4 - 187.0
40-49 years	84.0 - 104.0	92.0 - 112.7	113.0 - 124.0	128.0 - 142.0	145.0 - 166.2	166.2 - 185.3	169.0 - 220.0
50-59 years	68.2 - 98.9	89.8 - 107.0	107.0 - 127.5	130.0 - 143.0	151.0 - 178.8	178.3 - 207.3	188.3 - 224.0
60-69 years	72.8 - 97.0	86.8 - 104.0	104.0 - 118.0	123.0 - 130.5	137.5 - 158.5	162.0 - 183.0	178.0 - 211.0
18-69 years	69.0 - 81.0	82.9 - 91.9	102.8 - 111.0	124.0 - 129.0	143.0 - 153.0	167.0 - 180.0	181.0 - 193.1
**Women**							
18-29 years	43.0 - 65.0	54.0 - 72.0	65.0 - 80.0	80.0 - 90.0	92.0 - 103.0	105.2 - 124.2	124.2 - 154.1
30-39 years	45.0 - 74.0	67.9 - 82.6	74.0 - 87.0	87.0 - 98.0	101.5 - 115.0	119.2 - 132.0	132.1 - 153.2
40-49 years	65.0 - 74.0	67.0 - 82.0	74.0 - 87.6	87.2 - 106.5	114.5 - 124.5	128.2 - 139.5	138.6 - 181.0
50-59 years	31.0 - 83.0	68.0 - 92.9	83.0 - 102.0	103.2 - 118.0	123.0 - 140.0	145.0 - 162.8	164.0 - 176.0
60-69 years	78.0 - 99.8	84.8 - 102.0	99.8 - 105.7	105.8 - 128.0	131.0 - 151.5	156.2 - 169.0	169.0 - 198.0
18-69 years	43.0 - 66.0	68.0 - 79.0	79.9 - 85.0	93.0 - 100.0	115.0 - 122.0	136.0 - 146.0	160.0 - 172.1
**Men + Women**							
18-69 years	71.0 - 78.0	81.9 - 86.0	98.0 - 103.0	121.0 - 125.0	140.0 - 149.0	165.0 - 175.0	180.0 - 188.0

C) HDL (mg/dl)							
**Men**	5th	10th	25th	50th	75th	90th	95th
18-29 years	29.0 - 41.0	33.0 - 43.0	43.0 - 46.0	47.0 - 51.0	53.0 - 61.0	61.0 - 71.1	64.2 - 75.1
30-39 years	26.0 - 32.9	29.0 - 36.0	36.0 - 41.0	43.0 - 49.0	50.2 - 58.0	58.0 - 77.1	64.1 - 85.1
40-49 years	26.0 - 34.0	30.9 - 36.0	36.8 - 41.8	43.5 - 50.5	52.0 - 57.0	57.1 - 79.0	65.0 - 96.0
50-59 years	33.0 - 35.0	34.0 - 35.0	35.0 - 44.0	46.0 - 53.0	55.0 - 63.5	63.2 - 77.1	67.2 - 85.0
60-69 years	26.0 - 34.0	28.0 - 36.0	36.0 - 43.0	45.0 - 52.0	54.2 - 64.0	64.0 - 72.3	69.0 - 89.0
18-69 years	29.0 - 33.0	33.0 - 35.0	39.0 - 42.0	46.0 - 50.0	55.0 - 58.0	65.0 - 71.0	71.0 - 80.0
**Women**							
18-29 years	35.0 - 46.0	39.9 - 52.0	46.0 - 54.0	54.0 - 60.0	62.5 - 68.0	71.0 - 80.0	80.0 - 88.0
30-39 years	31.0 - 40.9	35.0 - 45.0	40.9 - 49.0	49.0 - 56.8	57.5 - 64.5	68.0 - 75.0	75.0 - 82.1
40-49 years	35.0 - 38.0	38.0 - 43.0	38.0 - 48.8	48.5 - 56.0	57.0 - 62.0	63.0 - 73.0	73.0 - 85.2
50-59 years	32.0 - 39.0	36.0 - 41.0	38.9 - 42.0	42.0 - 48.0	53.0 - 60.0	63.0 - 75.0	75.0 - 84.4
60-69 years	34.0 - 39.0	36.9 - 43.9	39.0 - 48.8	48.5 - 55.0	55.0 - 62.0	64.0 - 73.0	73.0 - 82.0
18-69 years	32.0 - 37.0	38.0 - 41.0	41.0 - 45.0	50.0 - 53.8	59.0 - 61.5	68.0 - 74.0	78.0 - 82.0
**Men + Women**							
18-69 years	33.0 - 35.0	36.0 - 38.0	43.0 - 46.0	53.0 - 56.0	63.0 - 66.0	75.0 - 79.0	80.1 - 85.0

D) TG (mg/dl)							
**Men**	5th	10th	25th	50th	75th	90th	95th
18-29 years	33.0 - 48.9	44.7 - 52.9	52.8 - 61.8	66.5 - 78.5	84.8 - 108.8	108.4 - 158.0	132.0 - 234.0
30-39 years	43.0 - 56.0	47.0 - 63.0	63.0 - 78.8	85.5 - 110.5	119.0 - 161.2	161.6 - 230.0	181.2 - 332.0
40-49 years	50.0 - 59.0	56.0 - 64.0	64.0 - 88.0	98.0 - 123.5	148.2 - 194.5	195.0 - 294.0	224.0 - 333.0
50-59 years	51.0 - 57.0	52.0 - 62.8	63.0 - 86.2	103.0 - 115.0	122.0 - 166.0	166.0 - 241.0	175.0 - 257.0
60-69 years	45.0 - 71.9	57.0 - 77.0	77.0 - 95.0	102.0 - 124.5	133.0 - 171.0	170.5 - 252.0	219.0 - 294.0
18-69 years	47.0 - 52.0	52.0 - 57.0	65.0 - 72.8	94.5 - 106.0	127.2 - 148.0	178.0 - 222.0	222.1 - 266.2
**Women**							
18-29 years	21.0 - 38.9	29.0 - 45.8	39.0 - 50.0	50.0 - 64.0	68.0 - 84.0	90.8 - 116.5	116.4 - 155.3
30-39 years	31.0 - 41.0	36.9 - 45.9	41.0 - 51.0	53.0 - 69.0	74.0 - 85.0	91.8 - 113.2	114.0 - 158.0
40-49 years	35.0 - 43.0	39.0 - 46.8	42.9 - 48.0	48.0 - 64.0	69.0 - 87.0	97.0 - 122.8	123.6 - 188.0
50-59 years	46.0 - 51.0	47.0 - 54.0	50.7 - 59.0	59.0 - 74.0	78.0 - 87.0	94.0 - 129.0	129.3 - 178.0
60-69 years	44.0 - 49.0	44.0 - 56.9	49.0 - 70.0	70.0 - 86.0	88.0 - 104.0	109.0 - 144.0	144.0 - 189.0
18-69 years	29.0 - 39.0	41.0 - 46.0	46.0 - 50.0	59.0 - 66.0	78.0 - 85.0	103.0 - 114.0	137.0 - 161.0
**Men + Women**							
18-69 years	44.0 - 47.0	48.0 - 53.0	63.0 - 69.0	85.0 - 93.0	117.0 - 129.0	163.3 - 181.0	194.0 - 237.0

**E) Lp(a) (nmol/L)**							
**Men**	5th	10th	25th	50th	75th	90th	95th
18-29 years	-	-	-	21.6 - 37.2	43.2 - 108.0	108.2 - 240.0	146.4 - 309.6
30-39 years	-	-	-	28.8 - 43.2	60.0 - 154.2	156.7 - 226.3	190.1 - 295.2
40-49 years	-	-	-	33.6 - 57.6	73.2 - 115.2	115.2 - 201.6	137.8 - 228.0
50-59 years	-	-	-	31.2 - 72.0	79.2 - 142.8	142.1 - 231.1	161.4 - 270.0
60-69 years	-	-	-	26.4 - 55.2	73.2 - 160.8	160.8 - 309.8	199.2 - 355.2
18-69 years	-	-	-	33.6 - 40.8	88.8 - 115.2	158.6 - 204.0	213.6 - 266.4
**Women**							
18-29 years	-	-	-	19.2 - 21.6	28.8 - 40.8	51.6 - 109.8	109.9 - 202.6
30-39 years	-	-	-	19.2 - 21.6	21.6 - 40.8	45.6 - 112.8	112.8 - 181.0
40-49 years	-	-	-	19.2 - 19.2	21.6 - 40.8	50.4 - 103.2	103.2 - 177.6
50-59 years	-	-	-	19.2 - 24.0	28.8 - 57.6	82.8 - 141.6	141.6 - 208.8
60-69 years	-	-	-	19.2 - 25.8	39.6 - 76.8	79.2 - 134.4	134.4 - 264.0
18-69 years	-	-	-	19.2 - 21.6	31.2 - 40.8	76.8 - 108.0	146.9 - 182.4
**Men + Women**							
18-69 years	-	-	-	33.6 - 40.8	84.6 - 108.6	160.8 - 187.4	204.1 - 247.2

**F) ApoB (mg/dl)**							
**Men**	5th	10th	25th	50th	75th	90th	95th
18-29 years	43.9 - 54.0	48.7 - 59.0	59.0 - 69.0	74.0 - 82.0	86.0 - 98.0	98.0 - 107.1	101.1 - 118.1
30-39 years	57.0 - 67.7	62.0 - 74.8	75.0 - 89.0	90.5 - 104.0	106.2 - 120.5	120.2 - 134.2	127.0 - 147.0
40-49 years	64.0 - 77.0	65.0 - 85.0	85.0 - 97.5	100.0 - 111.0	112.0 - 127.5	127.7 - 143.4	134.2 - 159.0
50-59 years	61.4 - 77.8	71.3 - 84.0	85.5 - 95.0	101.0 - 107.0	112.2 - 133.0	133.1 - 151.0	143.0 - 161.0
60-69 years	66.0 - 76.0	72.0 - 85.8	85.8 - 96.0	100.0 - 108.0	110.2 - 129.0	129.0 - 147.0	136.1 - 157.1
18-69 years	55.0 - 63.0	63.0 - 69.8	78.0 - 86.0	96.0 - 101.0	110.0 - 117.0	129.1 - 138.0	138.0 - 151.0
**Women**							
18-29 years	37.0 - 51.8	44.0 - 56.8	51.9 - 62.7	62.5 - 69.8	72.0 - 80.0	83.0 - 97.0	97.0 - 117.0
30-39 years	40.0 - 57.0	49.9 - 63.0	57.0 - 67.9	67.8 - 76.8	78.0 - 87.0	90.0 - 100.0	100.0 - 118.0
40-49 years	48.0 - 61.0	54.0 - 67.0	61.0 - 69.0	69.0 - 83.0	87.0 - 92.0	95.0 - 103.2	103.9 - 135.0
50-59 years	21.0 - 65.6	53.0 - 67.9	65.0 - 69.0	72.8 - 85.0	86.0 - 102.0	106.2 - 117.0	117.0 - 128.1
60-69 years	45.0 - 73.0	58.4 - 77.0	73.9 - 82.8	82.8 - 92.8	97.0 - 108.0	111.0 - 124.0	124.0 - 147.2
18-69 years	39.8 - 51.0	53.0 - 61.0	61.0 - 67.0	71.0 - 77.0	86.0 - 90.0	101.0 - 108.0	117.0 - 124.0
**Men + Women**							
18-69 years	55.0 - 61.0	63.0 - 66.0	75.0 - 79.0	90.0 - 95.0	107.0 - 111.0	124.0 - 131.0	134.0 - 143.0

G) ApoA1 (mg/dl)							
**Men**	5th	10th	25th	50th	75th	90th	95th
18-29 years	99.8 - 116.8	110.0 - 119.0	119.5 - 127.0	130.0 - 139.0	142.0 - 155.2	155.2 - 167.1	159.3 - 177.0
30-39 years	96.7 - 112.6	104.0 - 116.0	116.0 - 123.0	127.5 - 139.0	142.2 - 157.0	157.1 - 179.0	168.0 - 199.0
40-49 years	103.0 - 118.6	107.6 - 122.0	122.0 - 130.0	134.0 - 148.0	151.2 - 163.0	163.0 - 208.0	169.0 - 221.0
50-59 years	103.9 - 116.0	111.0 - 122.9	123.0 - 134.8	140.0 - 153.5	158.2 - 171.0	171.0 - 185.8	182.1 - 198.6
60-69 years	105.0 - 117.0	110.5 - 123.0	123.0 - 135.0	141.0 - 152.0	153.5 - 169.0	169.0 - 187.6	177.0 - 202.0
18-69 years	104.0 - 110.0	111.0 - 116.9	122.8 - 127.0	135.0 - 142.0	154.0 - 159.0	168.0 - 178.0	181.0 - 193.0
**Women**							
18-29 years	107.0 - 123.0	115.8 - 132.0	123.0 - 139.0	139.0 - 155.8	163.0 - 178.0	182.0 - 199.5	200.0 - 222.4
30-39 years	105.0 - 119.9	108.0 - 131.0	119.6 - 137.8	138.0 - 152.8	157.0 - 169.0	177.8 - 194.0	194.2 - 218.0
40-49 years	103.0 - 112.9	103.9 - 123.7	113.0 - 133.0	133.0 - 146.0	152.0 - 163.0	168.0 - 196.2	196.4 - 229.4
50-59 years	84.0 - 122.9	110.0 - 128.9	123.0 - 131.0	131.0 - 141.0	146.0 - 161.5	165.0 - 187.0	187.6 - 211.0
60-69 years	109.0 - 125.8	115.0 - 133.8	125.5 - 138.8	138.5 - 150.8	154.0 - 168.5	173.0 - 182.0	182.0 - 197.0
18-69 years	102.8 - 110.0	113.0 - 123.0	124.0 - 131.0	139.0 - 145.0	156.5 - 164.0	180.0 - 187.2	198.0 - 208.1
**Men + Women**							
18-69 years	109.0 - 114.0	116.0 - 120.0	130.0 - 134.0	148.0 - 152.0	167.0 - 174.0	190.1 - 197.1	202.0 - 212.0

H) non-HDL-C (mg/dl)							
**Men**	5th	10th	25th	50th	75th	90th	95th
18-29 years	61.0 - 79.0	72.7 - 88.7	88.8 - 101.0	108.5 - 122.0	127.0 - 146.0	146.0 - 163.1	154.1 - 186.0
30-39 years	86.9 - 106.0	91.0 - 112.9	112.8 - 132.0	138.0 - 152.0	157.0 - 183.0	183.2 - 202.6	194.1 - 213.3
40-49 years	93.0 - 118.0	108.0 - 126.0	126.0 - 147.0	150.0 - 166.0	170.5 - 188.8	187.8 - 226.0	198.9 - 235.0
50-59 years	80.1 - 110.8	101.8 - 130.8	129.0 - 142.0	148.0 - 159.0	164.0 - 199.5	199.2 - 236.4	212.8 - 243.0
60-69 years	85.9 - 108.0	104.0 - 121.0	121.0 - 140.0	142.0 - 158.0	163.0 - 187.8	188.3 - 215.0	206.0 - 242.0
18-69 years	82.0 - 91.0	91.0 - 104.0	117.0 - 126.0	142.0 - 148.0	164.0 - 176.2	195.0 - 206.0	208.0 - 231.1
**Women**							
18-29 years	58.0 - 76.0	59.0 - 84.0	76.0 - 90.0	90.0 - 102.8	105.0 - 119.0	122.0 - 146.0	146.2 - 171.3
30-39 years	59.0 - 82.9	73.0 - 90.0	82.9 - 101.7	101.5 - 111.5	117.0 - 131.0	136.5 - 151.2	151.3 - 176.4
40-49 years	78.0 - 87.9	79.0 - 92.0	88.0 - 100.0	100.0 - 116.8	127.5 - 137.0	141.2 - 166.2	166.5 - 204.0
50-59 years	49.0 - 99.5	69.0 - 104.0	98.7 - 114.0	114.0 - 129.8	137.0 - 153.5	158.0 - 169.8	169.4 - 192.3
60-69 years	89.0 - 111.0	103.3 - 116.0	111.0 - 120.9	120.8 - 141.2	148.0 - 169.0	175.8 - 188.0	188.3 - 226.0
18-69 years	58.9 - 77.0	79.0 - 88.0	89.0 - 99.0	106.0 - 114.0	129.5 - 136.5	153.2 - 162.0	178.0 - 187.1
**Men + Women**							
18-69 years	82.0 - 89.0	91.0 - 100.0	111.0 - 117.0	137.0 - 142.0	160.0 - 167.0	186.1 - 197.0	202.0 - 220.1

I) sdLDL-C (mg/dl)							
**Men**	5th	10th	25th	50th	75th	90th	95th
18-29 years	6.3 - 12.2	9.3 - 12.7	12.7 - 16.8	17.8 - 21.6	22.7 - 28.9	28.9 - 36.0	32.0 - 43.9
30-39 years	12.2 - 17.0	15.1 - 19.1	19.1 - 24.3	25.9 - 32.4	35.0 - 43.7	43.7 - 62.5	50.6 - 67.1
40-49 years	14.5 - 20.1	17.6 - 23.2	23.0 - 27.5	31.5 - 38.8	40.7 - 48.5	48.1 - 65.7	58.3 - 77.7
50-59 years	15.5 - 21.8	16.2 - 22.8	22.9 - 27.2	27.6 - 40.2	42.1 - 55.2	55.0 - 72.2	62.6 - 82.6
60-69 years	14.4 - 18.7	16.1 - 21.6	21.6 - 27.3	28.7 - 35.8	38.2 - 48.8	48.8 - 56.5	53.9 - 63.3
18-69 years	12.3 - 14.8	15.2 - 17.0	20.1 - 22.3	27.5 - 30.8	38.7 - 43.0	53.0 - 58.3	58.9 - 70.2
**Women**							
18-29 years	5.8 - 9.1	6.9 - 10.5	9.1 - 12.1	12.1 - 15.9	17.9 - 21.6	23.9 - 31.2	31.1 - 41.4
30-39 years	3.3 - 10.0	7.2 - 13.0	10.0 - 14.7	14.7 - 18.4	19.3 - 24.4	26.4 - 32.2	32.0 - 42.1
40-49 years	10.3 - 13.4	10.6 - 15.1	13.4 - 16.6	16.6 - 19.3	20.6 - 25.9	27.3 - 37.2	37.2 - 51.8
50-59 years	5.0 - 14.9	14.4 - 15.8	14.9 - 18.4	18.4 - 23.1	23.7 - 28.4	31.4 - 35.5	35.7 - 40.2
60-69 years	9.5 - 17.1	13.4 - 19.4	17.1 - 20.7	20.8 - 24.5	25.3 - 30.1	30.9 - 39.1	39.3 - 49.4
18-69 years	5.0 - 9.2	10.2 - 13.0	13.1 - 15.1	17.2 - 18.9	22.8 - 25.1	29.7 - 33.4	38.8 - 41.6
**Men + Women**							
18-69 years	11.3 - 13.2	14.4 - 15.5	18.7 - 20.0	25.2 - 27.0	34.6 - 37.6	44.4 - 51.3	54.2 - 60.9

J) VLDL (mg/dl)							
**Men**	5th	10th	25th	50th	75th	90th	95th
18-29 years	6.6 - 9.8	8.9 - 10.6	10.5 - 12.4	13.3 - 15.7	17.0 - 21.8	21.7 - 31.6	26.4 - 46.8
30-39 years	8.6 - 11.2	9.4 - 12.6	12.6 - 15.8	17.1 - 22.1	23.8 - 32.2	32.3 - 46.0	36.2 - 66.4
40-49 years	10.0 - 11.8	11.2 - 12.8	12.8 - 17.6	19.6 - 24.7	29.7 - 38.9	39.0 - 58.8	44.8 - 66.6
50-59 years	10.2 - 11.4	10.4 - 12.6	12.6 - 17.2	20.6 - 23.0	24.4 - 33.2	33.2 - 48.2	35.0 - 51.4
60-69 years	9.0 - 12.0	9.4 - 14.4	14.4 - 18.3	20.2 - 23.7	25.6 - 33.0	33.0 - 45.2	38.3 - 55.6
18-69 years	9.2 - 10.4	10.4 - 11.4	12.9 - 14.4	18.8 - 21.2	25.2 - 29.3	35.4 - 43.6	43.8 - 51.5
**Women**							
18-29 years	4.2 - 7.8	5.8 - 9.2	7.8 - 10.0	10.0 - 12.8	13.6 - 16.8	18.1 - 23.3	23.3 - 31.1
30-39 years	6.2 - 8.2	7.4 - 9.2	8.2 - 10.2	10.6 - 13.8	14.8 - 17.0	18.3 - 22.7	22.8 - 31.6
40-49 years	7.0 - 8.6	7.8 - 9.4	8.6 - 9.6	9.6 - 12.8	13.8 - 17.4	19.4 - 24.5	24.7 - 37.6
50-59 years	9.2 - 10.2	9.4 - 10.8	10.1 - 11.8	11.8 - 14.8	15.6 - 17.4	18.8 - 25.8	25.9 - 35.6
60-69 years	8.8 - 9.8	8.8 - 11.4	9.8 - 14.0	14.0 - 17.2	17.6 - 20.8	21.8 - 28.8	28.8 - 37.8
18-69 years	5.8 - 7.8	8.2 - 9.2	9.2 - 10.0	11.8 - 13.2	15.6 - 17.0	20.6 - 22.8	27.4 - 32.2
**Men + Women**							
18-69 years	8.8 - 9.4	9.6 - 10.6	12.6 - 13.6	17.0 - 18.6	23.2 - 25.6	32.4 - 35.8	38.2 - 45.8

For cells with '–' it was not possible to calculate the percentile due to Lp(a) detection limit, but it would be ≤20 nmol/L. TC, total cholesterol; LDL-C, low-density lipoprotein cholesterol; HDL-C, high-density lipoprotein cholesterol; TG, triglycerides; Lp(a), lipoprotein (a); apoB, apolipoprotein B; apoA1, apolipoprotein A1; non-HDL-C, total cholesterol minus high-density lipoprotein cholesterol; sdLDL-C, small dense low-density lipoprotein cholesterol; VLDL, very low-density lipoprotein cholesterol.

Supplementary Table S2. Prevalence of CVD in the Portuguese population, by sex and age.

	CVD	CI 95%
**Total**	**5.3%**	4.1% - 6.5%
**Men**	**5.8%**	4.1% - 7.4%
**Women**	**4.9%**	3.1% - 6.7%
**18-29 years**	**0.0%**	-
**30-39 years**	**0.4%**	-0.2% - 1.1%
**40-49 years**	**3.4%**	0.6% - 6.3%
**50-59 years**	**6.8%**	2.4% - 11.1%
**60-69 years**	**11.1%**	7.5% - 14.7%
**70-79 years**	**14.3%**	10% - 18.5%

CI, confidence interval; CVD, cardiovascular disease.
